# Image-based search and retrieval for biface artefacts using features capturing archaeologically significant characteristics

**DOI:** 10.1007/s00138-016-0819-x

**Published:** 2016-12-10

**Authors:** Mark Eramian, Ekta Walia, Christopher Power, Paul Cairns, Andrew Lewis

**Affiliations:** 1 Department of Computer Science, University of Saskatchewan, Saskatoon, SK Canada; 2 Department of Computer Science, University of York, York, UK

**Keywords:** Image retrieval, Image-based search, Biface, Archaeology, Artifacts, Flint

## Abstract

Archaeologists are currently producing huge numbers of digitized photographs to record and preserve artefact finds. These images are used to identify and categorize artefacts and reason about connections between artefacts and perform outreach to the public. However, finding specific types of images within collections remains a major challenge. Often, the metadata associated with images is sparse or is inconsistent. This makes keyword-based exploratory search difficult, leaving researchers to rely on serendipity and slowing down the research process. We present an image-based retrieval system that addresses this problem for biface artefacts. In order to identify artefact characteristics that need to be captured by image features, we conducted a contextual inquiry study with experts in bifaces. We then devised several descriptors for matching images of bifaces with similar artefacts. We evaluated the performance of these descriptors using measures that specifically look at the differences between the sets of images returned by the search system using different descriptors. Through this nuanced approach, we have provided a comprehensive analysis of the strengths and weaknesses of the different descriptors and identified implications for design in the search systems for archaeology.

## Introduction

It has long been recognized that the process of archaeology is by necessity a destructive one. In 1908, Sayce [31] echoed the sentiment of Flinders Petrie when he commented that “Scientific excavation means, before all things else, careful observation and record of every piece of pottery, however apparently worthless, which the excavator disinters”.

The very process which enables an archaeologist to understand each layer of an excavation requires the previous layer to be removed. The process of excavation is an unrepeatable operation [6]. For this reason, modern archaeology relies heavily on the use of recording technology to retain as much information as possible. One of the most common recording methods for in situ and excavated artefacts is that of digital photography.

Modern archaeological studies generate thousands of digital photographs of archaeological artefacts which are often assembled into large public archives such as those provided by the British Archaeology Data Service [1]. Archaeologists utilize such databases in identification and classification of newly discovered artefacts, training new archaeologists, and in public engagement with archaeological history. Currently, these archives are limited in their ability to allow archaeologists to search for relevant images related to a particular task. Search is often limited to keyword searches, or, at best, faceted browsing, which is problematic when image metadata is incomplete or in situations where the user is unfamiliar with the metadata schemes that are used in a particular archive.

The *Digging into Archaeological Data: Image Search and Markup (DADAISM)* project [12] is aimed at developing advanced computing technology, including image processing, to provide more useful and usable interactive systems for archaeologists to work with image archives. This paper reports on a major component of this project: a content-based image retrieval system for images of biface artefacts. This system uses image features to match a query image to images in the database with similar properties. The image features were developed with input from research archaeologists so that the image features capture properties of bifaces that would be used during classification tasks. This paper reports on the collection of data from archaeologists, the development of the image-based search algorithm, and an evaluation study to validate the results against existing classifications already present in the image metadata.

### Related work

The image analysis and pattern recognition literature contain several works related to feature extraction, similarity grouping, and classification of archaeological artefacts.

As part of Graphically Oriented Archaeological Database project, the authors in [21] presented an automatic method for shape extraction from raster images of line drawings to facilitate matching and retrieval. Smith et al. [32] developed a scheme to classify thin-shell ceramics based on colour and texture descriptors in order to aid in vessel reconstructions, using a new feature based on total variation geometry along with SIFT (scale-invariant feature transform). Abadi et al. [2] present a system for automatic texture characterization and classification of ceramic pastes, fabrics, and surfaces. They use Gabor filter along with linear discriminant analysis and k-nearest neighbour techniques in order to achieve the desired objectives. In [5], the authors analyse surface properties in pottery and lithic artefacts. They define texture with attributes such as coarseness, contrast, directionality, line-likeness, regularity, and roughness. Durham et al. [13] use generalized Hough transform as a practicable and robust tool for matching whole and partial artefact shapes.

Few examples of image-based identification systems for archaeological artefacts also exist in the literature. The work in [34] illustrates the use of computer vision techniques for the development of content-based image retrieval system for historic glass and an automatic system for mediaeval coin classification. In another work by [36] a prototype is presented which allows the end-user to search for similar digital library objects based on the image content. An integrated content and metadata-based retrieval system for art images in the domain of museum and gallery image collections is presented in [22]. Image retrieval methods are proposed to perform query on the basis of subimages. It also presents methods of querying by very low-quality images. IBISA [26] is a software tool that allows the user to perform image-based searches on the database of digital images of archaeological objects such as ancient coins. This system performs image segmentation with a method based on active contours and image registration with Fourier–Mellin Transform and computes similarity with classic intercorrelation factor. An extension of this system which is robust to lighting conditions [25] has also been proposed recently. CLAROS [10] allows image-based searches for sculpture and pottery images based on scale-, illumination-, and viewpoint-invariant image patches encoded using a bag-of-words scheme.

While the use of images, either hand drawings or photographs, remains the most common visual record of found lithics artefacts, there are opportunities to use 3D scanning technology to improve various aspects of archaeological work. For example, Grosman et al. [15] proposed the use of 3D scanning for purposes of documenting the essential characteristics of lithics in a standardized way that is less open to interpretation and error. Lin et al. [23] proposed the use of 3D scanning for analysis of lithic artefacts by using precise measurements of the cortex recorded in scans to make approximations of the size of artefacts. Finally, there are examples of people using 3D scanning for more specific types of analysis, such as Evans and Donahue [14] who looked at microwear on lithic artefacts.

Even with these advances in 3D scanning, there are currently still many thousands more photographs taken of artefacts than there are of 3D scans. Financial and time resource costs are one reason for this, as is the need to capture artefacts at different stages, including on site where scanners are unlikely to be available. In other cases, it is that the 3D scans provide some information, while images can provide different perspectives on the objects, such as the original patination on the surfaces of flint bifaces, and thus both are likely to be in ongoing use [8]. Combining this with the thousands of images that are already in archives worldwide, it is the case that archaeologists are going to need to find and work with images for the foreseeable future. The purposes for working with these images are varied; however, often it is to compare and identify objects to understand their function in society. These, and other purposes for using images in archaeology, are discussed in the contextual inquiry study described in Sect. 3.

With our work, we have expanded the corpus of image-based search of archaeological images to biface artefacts using image features based on established archaeological methodology for identifying similarities between the artefacts. Expert-verified metadata is used to validate the retrieval performance.

## Materials and methods

### Data set

**Fig. 1:**
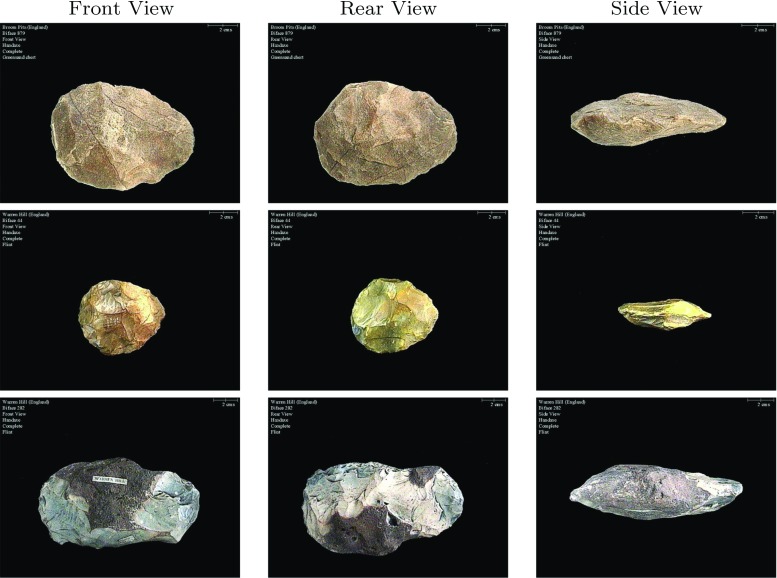
Example front, rear, and side views of four bifaces. Each *row* contains different views of the same artefact

Our data set is a public database from the British Archaeology Data Service consisting of 3501 images, each $500\times 375$ pixels in size, of 1167 biface artefacts in three views: front, rear, and side [27]. Figure 1 contains several examples of bifaces of varying size, shape, and texture, with different views of the same artefact shown in the same row. The images were captured at varying resolutions indicated by the scale bar on the top-right corner of each image.

The data set also consists of 52 metadata fields for each artefact including the site and country from where the biface was excavated, the artefact’s raw material, type, and its physical dimensions. The metadata was verified by biface experts and is of high quality, with little to no missing information. We utilized this information to define and measure relevance of results retrieved by our proposed system.

### Image features for biface search

Image-based features suitable for representing the characteristics of bifaces that are important for identification and classification were selected after conducting a contextual inquiry (CI) study in which research archaeologists with relevant experience were interviewed. The CI study and its outcomes are described in Sect. 3. The specific features that were selected based on the result of CI study are described in Sects. 4.2 and 4.3.

### Image-based search algorithm

An algorithm for identifying biface images similar to that of a query image based on the selected image features was developed. The details of this algorithm are presented in Sect. 5.

### Evaluation of image-based search algorithm

The biface search algorithm was validated in several ways by comparing the metadata of the images returned by the search algorithm to the metadata of the query image and by looking at patterns of disagreements in the retrieval results for different image descriptors. The evaluation methodology and results of the evaluation are described in Sect. 6.

## Biface classification contextual inquiry study

We conducted a contextual inquiry (CI) study to gain insight into how archaeologists judge similarity of bifaces. CI is a qualitative method developed by Beyer and Holtzblatt [7] to help design interactive systems that support users in their tasks. A CI study traditionally will have participants and researchers working in a collaborative way to explore different tasks *in situ*. In this case, we examined tasks where researchers were using image archives for research purposes.

### Participants

Four participants were recruited through mailing lists and personal contacts available to the Archaeological Data Service (ADS) at the University of York. Each participant had over 5 years of experience as a research archaeologist, made extensive use of picture archives in their work, and previously worked on prehistory sites with artefacts that included biface artefacts.

### Materials

Interviewees were provided with an information sheet that discussed how the interview would proceed and were provided with an informed consent form. Interviews were recorded on a Panasonic HD video camera, and the interviewer took notes to support analysis of the recording. Of particular interest to the interviewer was the documentation of explicit task flows and tacit knowledge the participant was using to make decisions. After the interview was completed, participants were provided with a demographic questionnaire through the online questionnaire tool Qualtrics.

During the interview, participants were shown some preliminary results of the image processing algorithms to give them some insight into how their data would be used. These images were printed in full colour at 150 dots per inch (dpi) to ensure clarity in the presentation.

### Procedure

Participants were met in a comfortable, quiet setting with a computer connected to the Internet. Participants were asked to bring with them any physical or digital materials that they use in typical, day-to-day work when trying to identify or classify artefacts.

Participants were asked about their own background in archaeology, their training, and their area of expertise. They were also asked what they considered to be the most important tasks that they undertook with online archaeological archives.

The CI study consisted of an initial semi-structured interview regarding how and when participants interacted with image archives and what they felt the most important aspects of the archives were. Further, the interview explored issues around favourite and least favourite aspects of working with image archives. This initial interview provided some initial context regarding what users do with their data.

Participants were asked about a recent time that they had used image archives to solve a research question and were asked to demonstrate how they used the archive to solve that problem. The participants demonstrated the tasks, describing how and why they used the archive. The interviewer would periodically stop the participant to discuss particular interesting aspects of the interaction, to elaborate on particular challenges or problems highlighted by the participant, or to clarify the purpose of particular actions. In this way, the interviewer was able to gain a better understanding of tacit aspects of the interaction that would not necessarily be raised during a traditional interview.

Finally, users were shown a set of images of bifaces and asked about different features that were used for classification and identification. This part of the interview led to a list of common features, as well as a set of research publications and secondary resources that would elaborate these features.

### Results

All participants were trying to find information about artefacts that related to the ones they were already working with. However, the final uses of the images were varied. A few researchers were working on presentations and displays for public engagement, while others worked to find images for inclusion in publications or lectures. However, the majority of the participants were trying to collect together sets of similar artefacts in order to identify an artefact they had in hand or, in some cases, to pass off that identification task to another specialist team member.

In these cases, there were often several types of analysis that would be conducted by other team members in parallel, with individuals contributing analyses from a number of perspectives. For example, if the artefact is longer than they are wide, it indicates that the artefact was, perhaps, the end product, whereas other lithics in an assemblage were potentially flakes from the manufacturing process. If the blade is heavily worked, meaning much of the stone has had the cortex removed, it gives indication of the advancement of the civilization that created the artefact. Finally, morphology will often indicate the function of the tool, with the visual characteristics providing a means of classifying the tools by its function in society [3, 28].

The recordings of the contextual inquiries were reviewed and partially transcribed to identify the tasks undertaken by participants in image archives, with a particular focus on the types of information they used to formulate and refine their queries. For purposes of this paper, we will focus on the characteristics of bifaces that emerged from this analysis. Experts reported that the following characteristics of biface tools are used to classify artefacts, judge the similarity of artefacts, and to formulate new queries for image search systems:

- *Blade versus flake* The artefact will be classified as a blade or a flake. Blades are twice as long as they are wide; flakes are less than twice as long as they are wide.
- *Worked versus unworked* A distinction is made between the cortex and core of the object. The cortex is the surface of the stone which has been worked by geological processes; the core is the interior part and is desired for tool working. As civilization is advanced, manufacture moved to using more of the core, while older artefacts exhibit more cortex. Artefacts may be distinguished by their type of *removal*: primary (removed from nearly unworked stone with substantial cortex remaining), secondary (a biface with only a thin seam of cortex created from stone closer to the core), or tertiary (flakes that have been created from the deep interior of the stone after primary and secondary strikes have removed the cortex and therefore exhibit a smooth, glassy quality).
- *Retouched versus non-retouched* Once a removal has been struck from the core, the piece will either be left as rubbish (because it is small or brittle), picked up and used as is, or retouched. Retouched pieces are worked into a specific shape for a specific task, e.g. knapping to give it a sharp edge. The manner of retouching was reported to be of significant interest to archaeological research.
- *Colour* The colour of stone bifaces from the same region may or may not be of a consistent colour and therefore may or not be diagnostic. Whether colour is of diagnostic use may depend on the data set and geographic regions under consideration.
- *Morphology* Morphology refers to the shape of the biface, or the number, shape, and length of individual tines on the artefact. It is the most common method of matching finds to a geographic region or culture. Complications arise when some morphological differences are derived from other morphologies; for example, a tine could have broken off and retouched into another type of biface through knapping.

In the next section we describe how different characteristics are mapped to image descriptors to match images automatically.

## Biface image descriptors

The CI study indicated that size, shape (morphology), and texture are diagnostic for bifaces. Our biface descriptor is a concatenation of image features capturing these three aspects. One set of morphological features capturing size and shape, and thirteen competing texture descriptors are described in the following sections. We begin with a description of the image preprocessing applied prior to extracting features.

### Image preprocessing

**Fig. 2:**
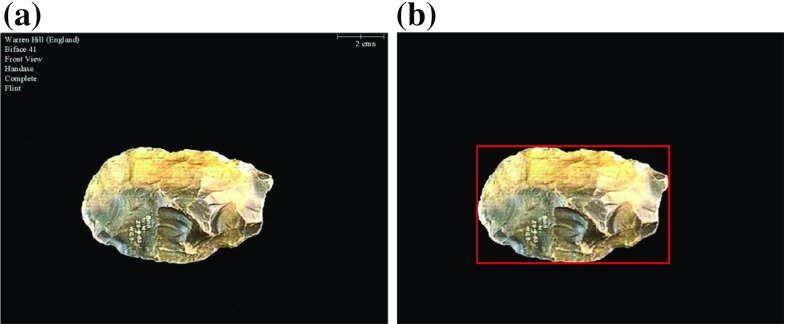
Preprocessing of images. Annotations and scale are removed, and bounding box of artefact determined

Biface images were preprocessed to isolate the object of interest from the rest of the image. A sample unprocessed image is shown in Fig. 2. Images were smoothed by using a $5\times 5$ Gaussian filter with $\sigma =0.5$ pixels. Images were then converted to the HSV colour space, and the biface object is segmented by selecting all pixels that have neither minimum nor maximum saturation. This gives accurate segmentation results for almost all bifaces, except for a very few which have very dark or very bright areas on their surface. To address this problem, all holes in the detected foreground regions were filled. The bounding box of the segmented artefacts was determined from their segmentations as in Fig. 2. Finally the subimages defined by the bounding boxes were converted from RGB colour to greyscale (luminance as defined by BT.601 standard). All features comprising the biface descriptor are extracted from these greyscale images.

### Morphological features

In this section we describe the size (geometric) and shape features that contribute to the biface descriptor.

#### Geometric features

Table 1 describes the geometric features extracted for a biface.

**Table 1:** Geometric features computed for the biface descriptor

Feature	Description
$g_1$	Scale-normalized length of the artefact’s bounding box
$g_2$	Scale-normalized width of the artefact’s bounding box
$g_3$	Scale-normalized area (number of pixels in the artefact’s segmentation)
$g_4$	The scale-normalized breadth of the artefact at 20% of the distance along the length of the bounding box
$g_5$	The scale-normalized breadth of the artefact at 80% of the distance along the length of the bounding box
$g_6$	The ratio $g_2/g_1$
$g_7$	The ratio $g_4/g_5$

Scale normalization was achieved by determining the image’s resolution in pixels per millimetre from the scale bar and converting distances and areas to units of mm and mm$^2$, respectively. We refer collectively to the features $\{g_1, g_2, \ldots , g_7\}$ as *g*.

#### Shape features

Biface shape is captured by a vector of Fourier descriptors. Following the centroid distance method of Zhang and Lu [38], the distance, *r*(*t*), between the centroid of the artefact and the *t*-th perimeter boundary point was computed. These distances are then encoded using the discrete Fourier transform:

1 $$\begin{aligned} {{\textit{FD}}_{l}} = \frac{1}{L}\sum _{t=0}^{L-1}{r(t) \exp \left( \frac{-j2\pi l t}{L}\right) }, \quad l=0,1,\ldots ,L-1.\nonumber \\ \end{aligned}$$

where *L* is the number of boundary points. A Fourier descriptor ${\textit{FD}}_{l}$ of a given shape is translation invariant and is made rotation invariant by retaining only the magnitude and discarding the phase information. For scale invariance, the magnitude of ${\textit{FD}}_{l}$ is divided by the DC component. The DC-normalized magnitude of the first 39 Fourier coefficients was retained for the shape description. Thus, the vector of Fourier descriptor features encoding a biface’s shape is:

2 $$\begin{aligned} {f} = \left\{ \frac{|{\textit{FD}}_{1}|}{|{\textit{FD}}_{0}|}, \frac{|{\textit{FD}}_{2}|}{|{\textit{FD}}_{0}|},\ldots , \frac{|{\textit{FD}}_{39}|}{|{\textit{FD}}_{0}|}.\right\} \end{aligned}$$

### Texture descriptors

The CI study indicated that texture of a biface’s surface appearance was an important indicator of the biface’s raw material type. In this section we describe 13 candidate texture descriptors. Some are existing features taken directly from existing literature, and others are new or are variations of existing features. The relative effectiveness of these 13 descriptors is evaluated in Sect. 6.

#### Uniform local binary patterns (ULBP)

Our ULBP descriptor consists of the 8-bit uniform local binary patterns as described in [33]. It is pertinent to mention here that we discarded the 59th bin containing non-uniform patterns as they have been observed to not be discriminating [33].

#### Orthogonal combination of linear binary patterns (OCLBP)

Our OCLBP descriptor consists of the features exactly as described by Zhu et al. [40].

#### Segmentation-based fractal texture analysis (SFTA)

Our SFTA descriptor uses segmentation-based fractal texture analysis [11] which decomposes the image into a set of binary images using a multithresholding scheme, and the fractal dimensions of the resulting regions describe the texture patterns. We used $n_{t}=8$ thresholds in our implementation. We did not use size (pixel count) and mean greylevel as suggested in [11] for feature vector construction because they are not discriminative for our data set. We only used the fractal dimension computed over different binary images resulting from the binary decomposition algorithm. Thus, we generated $2n_{t}$ features using the box-counting algorithm for $n_{t}$ thresholds, for a total of 16 features.

#### Global phase congruency histogram (GPCH)

Phase congruency (PC) is a measure of feature significance that is particularly robust to changes in illumination and contrast [19]. PC is based on the fact that the Fourier components are all in phase at the locations of significant features, such as step edges. It is a dimensionless quantity for measuring the consistency of local phase over different scales. It has been successfully used as feature in applications such as finger-knuckle-print recognition [39] and pose estimation for face recognition [30].

To compute a local PC map, a bank of quadrature-pair log-Gabor wavelets is applied to the image. Using the author’s MATLAB implementation [20] of the method in [19], we used $n=3$ scales of log-Gabor wavelet and 6 orientations $\theta = \{0,\frac{\pi }{6}, \frac{2\pi }{6}, \frac{3\pi }{6}, \frac{4\pi }{6}, \frac{5\pi }{6}\}$. We then computed a global phase congruency histogram (GPCH) by dividing the range of possible PC values, [0.0, 1.0], into 10 equal subintervals and counting the number of PC values in the PC map falling into each subinterval. The result is a vector of 10 texture features.

#### Angular radial phase congruency histogram (ARPCH)

We generated PC maps as in the previous section and subdivided the maps into radial sectors using the angular radial partitioning (ARP) described in [9]. The application of this partitioning scheme to PC maps is novel. ARP overlays on the PC maps *P* concentric circles which are divided into *Q* angular partitions. This gives $P \times Q$ sectors on the PC map.

A histogram of PC values is constructed for each sector of the PC map. We divided the range of possible PC values [0.0, 1.0] into *B* equal-sized subintervals and created a histogram $H_{s}$ for each sector *s* of an image as follows:

3 $$\begin{aligned} {H_{s}}(b)= \frac{{{\mathrm {count}}_{\mathrm {b}}({\textit{PC}}(s))}}{{\mathrm {area}}(s)},\quad b = 1, 2,\ldots ,B, \end{aligned}$$

where *B* is the number of histogram bins, ${\textit{PC}}(s)$ is the multiset of phase congruency values in the sector *s*, and ${\mathrm {count}}_{\mathrm {b}}({\textit{PC}}(s))$ is the number of elements in ${\textit{PC}}(s)$ which fall into bin *b*.

**Fig. 3:**
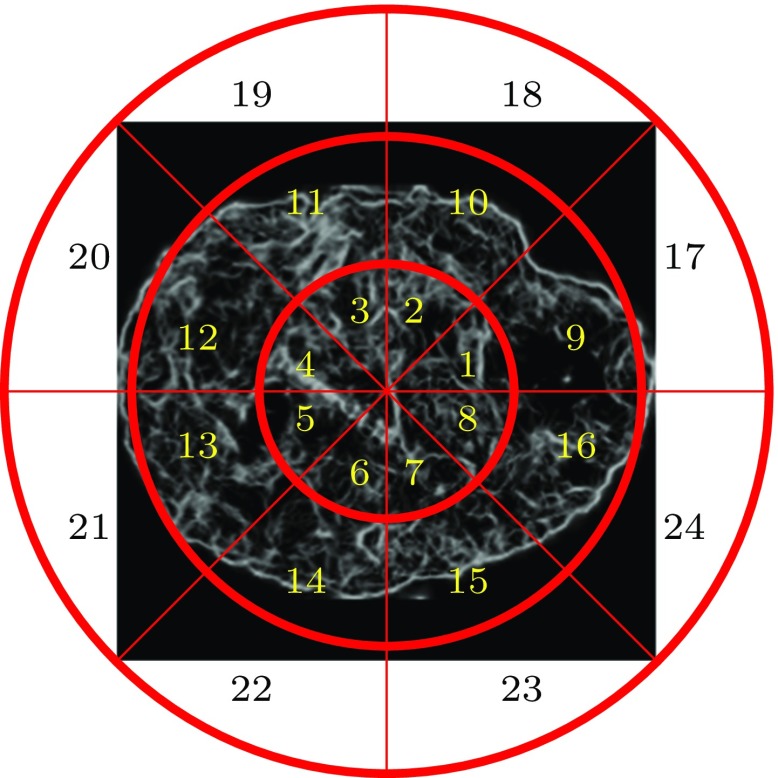
The radial and angular divisions of the PC map for $P=3$ and $Q=8$. *Numbers* indicate the sector ordering used when concatenating sector histograms

We then construct vectors $A_i$ by concatenating the values of the *i*-th bin of each sector histogram: $A_i = (H_1(i), H_2(i), \ldots , H_S(i))$. These are concatenated into a feature vector $A = (A_1, A_2, \ldots , A_B)$. We used $S=P\times Q=3\times 8 = 24$ sectors as shown in Fig. 3, and $B=10$ histogram bins, resulting in 240 features in *A*.

To reduce size of this descriptor, *A* is subdivided into subvectors $\hat{A}_j$ of length *S* / 2. We then perform singular value decomposition on each $\hat{A}_j$:

4 $$\begin{aligned} {\hat{A}_{j(1\times S/2)}= {U_{j(1\times k)}\varSigma _{j(k\times k)}V_{j(k\times S/2)}^T}}. \end{aligned}$$

Since $k = \min (1,S/2) = 1$, $\varSigma _j$ is a $1\times 1$ matrix, regardless of *S*, and we represent each subvector $\hat{A}_j$ by the singular value $\varSigma _j$. These are concatenated to form our 20-feature ARPCH descriptor of $(\varSigma _1,\varSigma _2,\ldots ,\varSigma _{2B})$.

#### Orientation-based phase congruency histograms (OPCH)

In this novel variation of PC-histogram-based features, instead of computing one PC map, as with the GPCH and ARPCH features, we computed eight separate PC maps for different filter orientations using [20]. We computed 8 different PC maps for the set of orientations $\theta =\{0,\frac{\pi }{8}, \frac{2\pi }{8}, \frac{3\pi }{8}, \frac{4\pi }{8}, \frac{5\pi }{8}, \frac{6\pi }{8}, \frac{7\pi }{8}\}$ using $n=4$ scales and took the summation over all scales *n* for each orientation in $\theta$.

We then generated the histogram of PC values for each direction in $\theta$:

5 $$\begin{aligned} {H_{\theta }}(b)= \frac{{{\mathrm {count}}_{\mathrm {b}}({\textit{PC}}_{\theta }(I))}}{{\mathrm {rows}}(I)\cdot {\mathrm {cols}}(I)}, \quad b = 1, 2,\ldots ,B \end{aligned}$$

where ${\mathrm {rows}}(I)$ and ${\mathrm {cols}}(I)$ are the dimensions of the bounding box found during image preprocessing. Again the range of PC values was divided into $B=10$ equal-size subintervals. The histograms for each PC map were concatenated to form our orientation-based phase congruency histogram (OPCH) descriptor consisting of 80 features.

#### Gabor wavelet features (GWF)

We extracted features from the Gabor wavelet transform of a artefact’s image as in [24]. A filter bank of Gabor wavelet functions with different orientations is convolved with the image to obtain a complex response $R_{s\alpha }(x,y)$ as follows

6 $$\begin{aligned} {R_{s\alpha }(x,y)}={\sum _{m}\sum _{n}I(x-m,y-n)\varPsi _{s\alpha }^*(m,n)} \end{aligned}$$

where *I* is the image; $\varPsi _{s\alpha }^*$ is the complex conjugate of $\varPsi _{s\alpha }$, which is a self-similar function generated from the dilation and rotation of the mother wavelet $\varPsi$. The definitions of $\varPsi$, $\varPsi _{s\alpha }$ and $\varPsi _{s\alpha }^*$ can be found in Eqs. 1–3 of [24] where they are called *g*, *G*, and $g_{mn}$, respectively. Intuitively *s* determines the scale of the filter, and $\alpha$ determines its orientation.

We chose six orientations and three scales for our implementation, obtaining 18 responses $R_{s\alpha }(x,y)$ corresponding to all combinations of $\alpha =0,1,\ldots , 5$ and $s=0,1,2$. As in [24], the means $\mu _{s\alpha }$ and standard deviations $\sigma _{s\alpha },$ of the magnitude of the 18 response images were used as features resulting in a descriptor of 36 features.

It is pertinent to mention that $\sigma _{x}$ and $\sigma _{y}$ from Equation 1 in [24] were computed as $\sigma _{x}={1}/{2\pi \sigma _{u}}$ and $\sigma _{y}={1}/{2\pi \sigma _{v}}$ where $\sigma _{u}$ and $\sigma _{v}$ are as in Equation 4 in [24]. The scale factor *a* ([24], Equation 3) is also computed in accordance with Eq. 4 of [24]. In this equation, the lower and upper frequencies of interest, $U_{l}$ and $U_{h}$, were chosen as 0.05 and 0.4, respectively.

#### Log-Gabor wavelet features (LGWF)

A variant of the GWF features defined in the previous subsection was derived by substituting log-Gabor wavelet filters [4] for the Gabor wavelet filters. Log-Gabor wavelets are represented in polar coordinates as follows:

7 $$\begin{aligned} {{\textit{LG}}(\rho ,\theta )}= {\exp \left( \frac{\left( -\ln \left( \frac{\rho }{\rho _{0}}\right) \right) ^2}{\left( 2\ln \left( \frac{\sigma _{\rho }}{\rho _{0}}\right) \right) ^2}\right) }{\cdot \exp \left( \frac{-(\theta -\theta _{0})^2}{2\sigma _{\theta }^2}\right) }, \end{aligned}$$

where ${\rho }$ is the radial coordinate, ${\theta }$ is the angular coordinate, and $\rho _{0}$ is the centre frequency of the filter, defined as $\frac{1}{\lambda }$ for wavelength ${\lambda }$, $\theta _0$ is the orientation of the filter, and $\sigma _{\rho }$ and $\sigma _{\theta }$ are the bandwidths for the radial and angular components.

We used three filter scales $\rho _0 = \{ 1/3, 1/6, 1/12 \}$ corresponding to wavelengths of 3, 6, and 12 pixels and six different orientations $\theta _{0}= \{\frac{\pi }{6}*(i-1) \mid i=1,\ldots ,6\}$. For each $\rho _0$, the radial bandwidth was selected so that ${\sigma _{\rho }}/{\rho _{o}}=0.65$, which corresponds approximately to 2 octaves. For all orientations, the angular bandwidth was set to two-third of the angular interval $\pi /6$ (the interval between selected filter orientations $\theta _0$) or $\pi /9$.

The LGWF features were then obtained by computing the mean $\mu _{\rho \theta }$, standard deviation $\sigma _{\rho \theta }$, and skewness $\gamma _{\rho \theta }$ of the magnitude of 18 responses of log-Gabor wavelet filters, defined as follows.

8 $$\begin{aligned} \mu _{\rho \theta }= & {} \frac{E(\rho ,\theta )}{MN} \end{aligned}$$

9 $$\begin{aligned} \sigma _{\rho \theta }= & {} \sqrt{\frac{\sum \sum {{(E(\rho ,\theta )-\mu _{\rho \theta })^2}}}{MN}} \end{aligned}$$

10 $$\begin{aligned} \gamma _{\rho \theta }= & {} \frac{1}{{\sigma _{\rho \theta }}^3}.{\frac{\sum \sum {{(E(\rho ,\theta )-\mu _{\rho \theta })^3}}}{MN}} \end{aligned}$$

where *M* and *N* are the number of rows and columns of *I*, and $E(\rho ,\theta ) = \Vert {\mathrm {idft}}({\textit{LG}}(\rho ,\theta )*{\mathrm {dft}}(I))\Vert$ denotes the magnitude of response image obtained by the pointwise multiplication of discrete Fourier transform of the image *I* with the log-Gabor wavelet filter. Functions ${\mathrm {dft}}$ and ${\mathrm {idft}}$ denote the forward and inverse discrete Fourier transforms, respectively. The combinations of 3 scales and 6 orientations and 3 features per combination yield a descriptor containing 54 features.

#### Binary texton features (BTF)

We used the “Binary-MR8” features exactly as described in [16]. These features were chosen since they do not require training. In brief, these features are computed by employing a bank of maximum-response filters consisting of some anisotropic filters at different orientations and scales and some radially symmetric filters. This is followed by a dimensionality reduction process which yields a texture descriptor of length 2048.

#### Fusion of LGWF and ARPCH (L–A)

LGWF and ARPCH focus on two different aspects of filter responses. The LWGF is based on the global statistics of log-Gabor response image, whereas the ARPCH is based on the local statistics of the phase congruency of the filters response. Various face recognition algorithms focus on the use of phase as well as magnitude-based features of Gabor responses [37] [29]. On the similar lines, considering the complementary (global vs. local) nature of LGWF and ARPCH features, we fused them to be used as a new texture descriptor L–A. Fusion was performed at the “score level” by computing the distance metric (Chi-square) for LGWF and ARPCH separately for each pair of images, normalizing these distances with the min–max normalization described previously, and taking the sum of the normalized distances as the distance between the query image and the database images.

#### Fusion of LGWF and BTF (L–B)

The filters used in the extraction of binary texton features (BTF) are sensitive to different image features compared to the log-Gabor wavelets used in LGWF. The filter bank used to compute BTF features is rotationally invariant and contains isotropic (Gaussian and Laplacian of Gaussian) as well as anisotropic derivative-based filters which aim to capture edges and bars at multiple orientations and scales and, thus, is well capable of encoding both rotationally invariant and oriented textures. BTF features are derived from the responses of MR8 filters, generating a binary texton for multiple orientations, in a fashion similar to rotation-invariant uniform LBP features. LGWF features are based on response statistics of the log-Gabor filter bank, which consists of log-Gabor filters that are defined in the log-polar coordinates of the Fourier domain as Gaussians shifted from origin, well capable of obtaining the localized frequency information and their zero response for a constant signal provides invariance to greylevel shift. Given the complementary nature of these features, we combined them to create a new texture descriptor L–B for raw material characterization. This fusion is implemented at the score level as described in Sect. 4.3.10.

#### Fusion of LGWF, ARPCH, and BTF (L–A–B)

We previously observed that LGWF is complementary to ARPCH and BTF. ARPCH and BTF are also complementary. ARPCH is derived from local phase congruency which is an illumination and contrast-invariant feature of an image, whereas BTF is based on anisotropic first and second derivatives, and both of them are based on two different broad approaches to feature analysis in human vision. The possibility of making human feature detection complete by combining insights from both the approaches is listed out in [17]. We fused all three of these descriptors into a new descriptor L–A–B, again using score-level fusion as described in Sect. 4.3.10.

#### Fusion of LGWF, ARPCH, and SFTA (L–A–S)

Finally, the complementary descriptors LGWF, ARPCH, and SFTA were combined using score-level fusion as in Sect. 4.3.10. This feature is abbreviated as L–A–S.

## Image-based search algorithm for bifaces

### Feature extraction

The shape descriptor *f*, the geometric features $g=\{g_1,\ldots , g_7\}$, and all thirteen texture descriptors described in Sect. 4.3 were computed for each image.

### Dissimilarity measures

The distance between each geometric feature of a query image *Q* and a database image *I* is defined as a simple absolute difference:

11 $$\begin{aligned} {D_{\mathrm {{geo}}}}^i(Q,I) = \left| \left( g_{i}^Q-g_{i}^I\right) \right| ,\quad i = 1,\ldots ,7, \end{aligned}$$

where $g_{i}^Q$ and $g_{i}^I$ represent the geometric features of objects in images *Q* and *I*, respectively.

The distance between shape descriptors of *Q* and *I* is defined as the $L_2$ norm:

12 $$\begin{aligned} {D_\mathrm {{shape}}}(Q,I)= \sum _{i=1}^{n_\mathrm {{shape}}}{\sqrt{\left( f_{i}^Q-f_{i}^I\right) ^2}}, \end{aligned}$$

where $f_{i}^Q$ and $f_{i}^I$ represent the shape features of images *Q* and *I*, respectively, and $n_\mathrm {{shape}}$ is the number of shape features, which, as previously noted, is 39.

The distance between texture descriptors of images *Q* and *I* is defined as the Chi-square distance:

13 $$\begin{aligned} {D_\mathrm {{texture}}}(Q,I)= \sum _{i=1}^{n_\mathrm {tex}}\frac{\left( {H_{i}^Q-H_{i}^I}\right) ^2}{H_{i}^Q+H_{i}^I}, \end{aligned}$$

where $H_{i}^Q$ and $H_{i}^I$ represent the features of the texture descriptors of images *Q* and *I*, respectively, and $n_\mathrm {{tex}}$ is the length of the texture descriptor.

The min–max normalization technique [18] is employed to separately normalize each of the geometric feature distances:

14 $$\begin{aligned} D_\mathrm {geo}^{\mathrm {norm}(i)}(Q,I)&= \frac{D_\mathrm {geo}^i(Q,I)-\min _k\left( D_\mathrm {geo}^i\left( Q,I_k\right) \right) }{\max _k\left( D_\mathrm {geo}^i\left( Q,I_k\right) \right) -\min _k\left( D_\mathrm {geo}^i\left( Q,I_k\right) \right) },\nonumber \\ i&= 1,2\ldots ,7 \end{aligned}$$

where the index *k* is over all images in the database. Shape and texture distances, $D_\mathrm {{shape}}$ and $D_\mathrm {{texture}}$, are normalized in the same way.
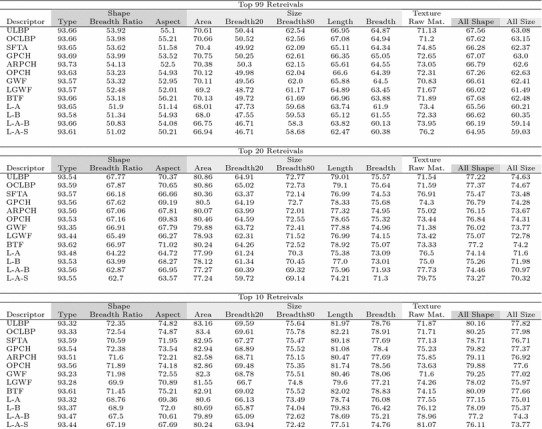


### Retrieval algorithm

Given a query image, the steps mentioned in Algorithm 1 are performed to retrieve similar images from the database. In order to avoid online computation, the geometric, shape, and texture descriptors of the objects in the database images are precomputed. The algorithm can be used with any one of the texture descriptors described in Sect. 4.3 together with the geometric features *g* and the shape descriptor *f*.

## Validation of the image-based search algorithm

This section discusses the performance evaluation of Algorithm 1.

### Validation methodology

For each texture descriptor, the 1167 front-view images were used in a leave-one-out style methodology. Each of the images was used once as the query image and the *N* most similar images were retrieved from the set of remaining images using Algorithm 1.

Section 2.1 briefly described the metadata associated with each image. Certain fields of this metadata were used to analyse the performance of Algorithm 1 by comparing the metadata of the retrieved images to the metadata of the query image. Many of the metadata fields contain information that is not useful for image-based matching because they do not directly describe appearance of the objects, for example, fields that detail the date and location of the find. The metadata fields chosen for the use in the analysis were motivated by the findings of the CI study detailed in Sect. 3. The *biface type* (e.g. hand axe) field was used due to its relationship with biface morphology and the blade vs. flake categorization. The *raw material type* field (e.g. flint or chert) was used because this is related to geographic location and availability of material at a site. Several metadata fields describing physical measurements of the artefacts were used since they can be used to judge how well the physical measurements of the bifaces are extracted from the images. These fields were: *Area*, *Aspect Ratio*, *Length*, *Breadth*, *Breadth at 20% of the length* (*Breadth20*, the breadth of the artefact at 20% of the distance between the wider end of the artefact to the narrower end of the biface), *Breadth at 80% of the length (Breadth80)*, and the ratio of *Breadth80* to *Breadth20*.

### Individual metadata match performance

As a first look at how the different texture descriptors performed, for each texture descriptor *t* and metadata field *m*, we computed the percentage of retrieved images over all queries where field *m* of the retrieved image matched field *m* of the query image. The results are shown in Table 2 for the 10, 20, and 99 most similar images retrieved by Algorithm 1. The biface type field was matched about 93% of the time for all texture descriptors; the lack of variability observed coincides with the expectation that texture descriptors do not capture information about biface type.

Different texture descriptors exhibited greater differences in ability to match raw material type ranging from about 71% for ULBP, OCLBP, and GWF to about 76% for L–A–S for the top 99 retrievals. Generally, the various descriptors consisting of descriptors fused with LWGF resulted in the most raw material type matches, with SFTA and ARPCH also performing well on their own. For 10 and 20 retrievals the same trends are present, though the spread increases to a low of about 72% to a high of about 81% for 10 retrievals. This indicates that the descriptors which are fusions with LWGF are more likely to return bifaces of the same raw material type with higher ranks.

Even though the texture descriptors are not capturing information about a biface’s physical dimensions, the trend was that ULBP, and OCLBP resulted in more matches for the physical measurement metadata fields, while the fusions of descriptors with LGWF performed the worst, though in all cases the difference between best and worst was only about four percentage points. This suggests that texture descriptors that are better at matching raw material type do so at the expense of finding bifaces of a more similar size, likely because bifaces of the same raw material type but with greater differences in physical dimensions are selected over bifaces of more similar physical dimensions. The last two columns of the table where we have aggregated the results for metadata features meant to be, respectively, captured by shape and size image features, and we can see the trade-off clearly. Texture descriptors that are better able to extract bifaces with the correct raw material type do so at the expense of matching objects of more similar shape and size.

**Table 2:** Percentage of queries for which each metadata field matched for each texture descriptor

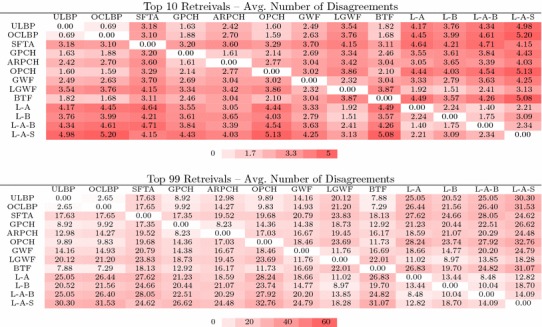	

The last two columns group the results for metadata meant to be captured by size and shape features, respectively

### Normalized accuracy

In order to get a better picture of the performance of the retrieval system we must recognize that, in general, it will not be possible to get very high percentages of metadata matches since there may not even exist sufficiently many relevant images with similar metadata. In this section we use a measure we call *normalized accuracy* which compares the number of metadata matches in the retrieved images to the highest number of metadata matches that it would be possible to achieve by retrieving the same number of images.

The relevance score, $rel_{{\mathrm {score}}}$, which characterizes the relevance of an image *D* retrieved by Algorithm 1 to the given query image *Q* is defined as:

15 $$\begin{aligned} {rel_{{\mathrm {score}}}(Q,D)}= \sum _{i=1}^{9}meta_{{\mathrm {score}}}^{i}(Q,D), \end{aligned}$$

where $meta_{\mathrm {score}}^{i}(Q,D)$ is the binary match score function

16 $$\begin{aligned} {meta_\mathrm {score}^{i}(Q,D)} =\left\{ \begin{array}{ll} 1,&{} \quad \text {if } meta^{i}(Q) = meta^{i}(D), \\ 0,&{} \quad \text {otherwise} \end{array}\right. \end{aligned}$$

where $meta^{i}(Q)$ and $meta^{i}(D)$ refer to the value of the $i^{th}$ metadata feature of *Q* and *D*, respectively. Thus, if the two images exactly match in all of their metadata features, the relevance score would be 9 and the image *D* is considered highly relevant to the query image *Q*.

The maximum possible total relevance score, denoted as $max_\mathrm {score}(Q)$, for a query image *Q* and *N* retrievals is determined by computing $rel_{\mathrm {score}}(Q,D)$ for every database image *D* and finding images $E_1, \ldots , E_N$ with the *N* largest relevance scores. Then $max_\mathrm {score}(Q) = \sum _{i=1}^{N} rel_{\mathrm {score}}(Q, E_i)$. Similarly, the actual total relevance score achieved by Algorithm 1 for query image *Q* is $query_{\mathrm {score}}(Q) = \sum _{i=1}^N rel_{\mathrm {score}}(Q, D_i)$, where $D_1, \ldots , D_N$ are the *N* images that were retrieved. Finally, the *normalized accuracy* for a given query *Q* is:

17 $$\begin{aligned} {Ret_\mathrm {accuracy}}(Q)= \frac{query_\mathrm {score}(Q)}{max_\mathrm {score}(Q)}. \end{aligned}$$

**Fig. 4:**
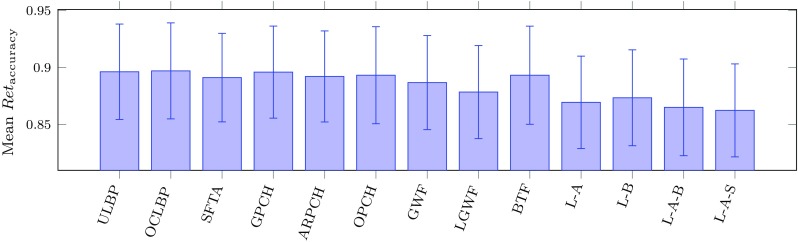
Mean and standard deviation of normalized accuracy for each texture descriptor over all queries using Algorithm 1, $N = 99$ retrievals

For each texture descriptor, the normalized accuracy was computed for each query using the leave-one-out methodology described in Sect. 6.1 at $N=99$ retrievals. The mean normalized accuracy for each texture descriptor over all queries is shown in Fig. 4. A Wilcoxon signed-rank test was performed pairwise on the texture descriptors to test the null hypothesis that the 1167 paired normalized accuracy scores came from the same distribution. In nearly all cases the null hypothesis was rejected at the $\alpha = 0.05$ level with $p < 0.0069$ with the exceptions of ULBP/GPCH ($p=0.0733$) and OPCH/BTF ($p=0.9236$). Results for $N=10$ and $N=20$ retrievals were extremely similar, both in the magnitude of the means for each descriptor and the results of the Wilcoxon signed-rank tests. These results indicate that all of the texture descriptors (along with the shape and geometric features) are doing very well overall in matching the most relevant images. The differences in performance, while small in magnitude, are statistically significant and indicate that ULBP, OCLBP, GPCH, and BTF are the best texture descriptors on average.

However, given that this image retrieval system is intended to support user search and reasoning over archives, looking only at differences in mean performance does not provide a sufficiently nuanced understanding of the differences between the algorithms and what the consequences of those differences might be for the search experience. In the next section, we propose an alternative analysis that examines the data sets from a differing perspective.

For the remainder of our analysis we will be interested in the behaviour of the ULBP, OLCBP, GPCH, and BTF as the group of descriptors that had the “best” overall performance in Table 4 which will refer to as the SO group (superior overall group) and the fused descriptors L–A, L–B, L–A–B, and L–A–S as the IO group (inferior overall group) which had the “worst” performance in Fig. 4.

### Pairwise symmetric difference comparisons

All of the texture descriptors resulted in similar overall accuracy (Fig. 4). When the overall accuracy score is within two to three percentage points, it is tempting to say that any particular algorithm would be suitable. However, because this algorithm is intended to support user search, these aggregate measures of accuracy do not give us any indication of what result sets look like which could be very important in choosing a particular algorithm. There could be distinct differences in the sets of images that are returned because when comparing any two descriptors across the nearly 1200 query images, there are a number of different distributions of results that could produce similar overall accuracy scores. If there are situations where one descriptor performs particularly well or particularly poorly this may skew the average up or down. Similarly, if the result sets retrieved by two different descriptors consistently share a number of results, this will obscure the actual differences between the algorithms.

To better elucidate the previously observed trade-offs apparent in the set of texture descriptors we performed an analysis in which, for a given query image *Q* and pair of texture descriptors $D_1$ and $D_2$, we only considered the symmetric difference of the images retrieved by Algorithm 1 using $D_1$ and $D_2$, that is, images not in common to both retrievals.

**Table 3:** Average size of the symmetric difference for the top 10 and top 100 retrievals

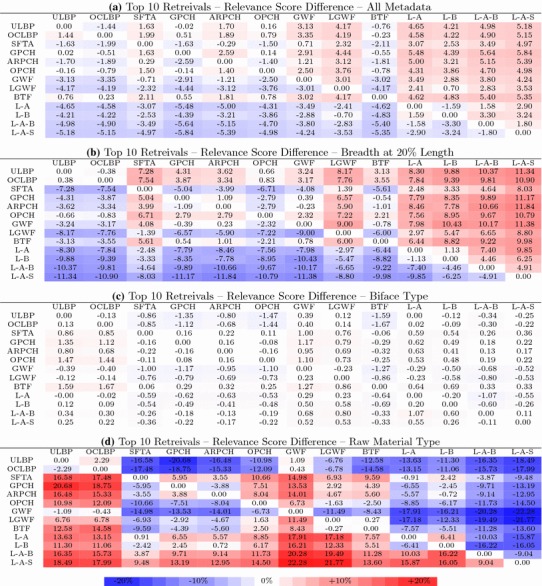	

For a pair of descriptors $D_1$ and $D_2$, let $R^1_q$ and $R^2_q$ be the set of images retrieved for a given query image *q* by Algorithm 1 using $D_1$ and $D_2$, respectively. Obtain the symmetric difference of $R^1_q$ and $R^2_q$: $\varDelta _q(D_1, D_2) = R^1_q\cup R^2_q \setminus R^1_q\cap R^2_q$. Let $\delta ^1_q(D_1,D_2)$ be the elements of $\varDelta _q(D_1, D_2)$ in $R^1_1$, and let $\delta ^2_q(D_1,D_2)$ be the elements of $\varDelta _q(D_1, D_2)$ in $R^2_q$.

#### Average number of disagreements

If two sets of results for a given query image using different descriptors are identical, then they are performing nearly the same for that image. For retrieved sets that are small (e.g. 10 retrievals), then it would be expected that most results would be shared. If two descriptors return largely the same set of images, then we can assume there is no major difference between them and that the descriptors are doing essentially the same thing. For larger retrieval sets, there are fewer appropriate matches that will occur, so it is valuable to examine which feature performs best after removing shared items.

Table 3 presents the results for each pair of descriptors for the top $N=10$ and $N=99$ retrievals. Looking closely at the results for the SO feature group, we see that these descriptors are all performing nearly identically in that the largest average difference between retrieval sets for pairs of descriptors in this group is 2.46 (GPCH vs. BTF). However, when comparing these 4 descriptors to those in the IO group, we find that there are substantial differences between the retrieval sets for the IO group and the SO group with ULBP/L–A–S producing the highest mean difference of 5.2 images between the sets.

When we increase to 99 retrievals we find similarities between the IO group and the SO group that are even more pronounced. Pairwise, all of ULBP, OCLBP, and GPCH produce result sets that have fewer than 13 different images on average. As a result, it is hardly worth calculating the differences in average accuracy in these algorithms as they are doing largely the same thing.

Comparing the SO group to the IO group we see a much more pronounced difference. OCLBP and L–A–S have 31.5 different images (nearly a third) in their result sets on average. Most pairs consisting of one IO and one SO group member average between 10 and 25 disagreements (10.1–25.3% of retrievals). These results show that on average, there are a non-trivial number of differences between the set of results returned by different descriptors, but these are masked in the overall accuracy result. Given these differences, it is worth looking closely at the differences, first by looking at the mean accuracy of the differences.

#### Differences in metadata matches for symmetric differences

We computed the difference in the percentage of metadata matches for all of the images in the symmetric differences of each query. The process is now explained in detail.

Let $m^1_q(D_1,D_2)$ be the number of metadata matches between the elements of $R^1_q$ and the query image *q*, and $n^1_q(D_1, D_2) = 9|R^1_q|$ (the total number of metadata fields associated with the elements of $R^1_q$). Symmetrically define $m^2_q(D_1,D_2)$ and $n^2_q = 9|R^2_q|$. Then compute:

18 $$\begin{aligned} P(D_1, D_2) = \frac{\sum _q m^1_q(D_1, D_2)}{\sum _q n^1_q(D_1, D_2)} - \frac{\sum _q m^2_q(D_1, D_2)}{\sum _q n^2_q(D_1, D_2)} \end{aligned}$$

which is the difference between the percentage of metadata matches in the symmetric difference of query results arising from descriptors $D_1$ and $D_2$. The two subtractive terms in Eq. 18 are relevance scores for $D_1$ and $D_2$, and we call $P(D_1,D_2)$ the *relevance score difference*.

**Table 4:** Relevance score difference comparisons for top 10 retrievals

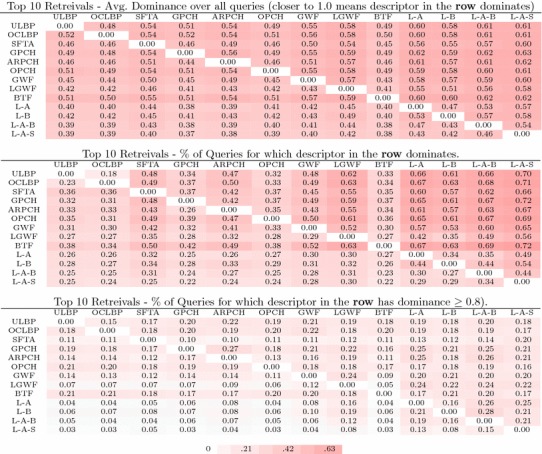	

Table 4a shows the relevance score difference for all pairs of texture descriptors for queries using Algorithm 1. The rows are indexed by $D_1$ and columns by $D_2$; thus, the top-right most entry indicates that the percentage of metadata fields matched was 5% more for ULBP than for L–A–S. This table confirms the observations from Table 2 that the descriptors in the SO group have the best overall performance (most entries in their rows are positive).

Table 4b shows the same relevance score difference but only considering the single metadata field *Breadth at 20% Length* (definitions of $m^1_q$, $m^2_q$, $n^1_q$, and $n^2_q$ are adjusted accordingly) rather than all metadata fields together. The results indicate that the SO group are generally overall strong performers for matching this metadata field. Results are similar for the other geometric features in *g*.

Table 4c shows the relevance score difference considering only the metadata field *biface type*. The data indicate that the choice of texture descriptor has very little effect on the matching of *biface type* which is consistent with the intuition that texture should not be indicative of the type of biface since this is primarily determined by shape.

Table 4d shows the relevance score difference considering only the metadata field *raw material type*. Here we see a dramatic difference in performance where the table colouring resembles an inversion of the other tables. The best performing descriptors in the other tables are among the worst performers for matching raw material type. The descriptors in the IO group are the best performers which is consistent with observations from Table 2.

This set of results demonstrates that the descriptors in the IO group are better at matching *biface type* at the expense of other metadata fields. The more pronounced differences in performance between descriptors observed in all of these tables, relative to Table 2, are because we are considering only images in the symmetric differences of the retrievals, that is, the query results for which two different descriptors disagree.

For the top 99 retrievals the results are similar to those for the top 10 retrievals.

#### Dominance

As we can see from the above results, there are distinct differences in the mean accuracy of the symmetric difference. However, like the overall accuracy calculations, these averages may be hiding nuances about the symmetric difference sets. If, for example, one descriptor consistently performs better in its results, then it could be argued to choose one over another. As a result, we considered the question of how often does one descriptor perform better than another descriptor which we will refer to as *dominance* which is calculated using the A-primed statistic [35].

For query image *q* and descriptors $D_1$ and $D_2$, we rank the elements of the symmetric difference of the query results $\varDelta _q(D_1, D_2)$ according to the number of metadata matches with the query image as they would be in a Mann–Whitney test. The image in $\varDelta _q(D_1, D_2)$ with the greatest number of metadata matches gets rank 1, the next most gets rank 2, and so on. In the case of ties, the tied images get the average of the rank they occupy, so if three images that occupy ranks 7, 6, 5, and 4 have the same number of metadata matches, they are all assigned rank 5.5. We then calculate the sum of these ranks for the images from $\varDelta _q(D_1, D_2)$ that are in $R_q^1$, which we previously named $\delta ^1_q(D_1, D_2)$ (see Sect. 6.4) and denote this as $ranksum$. Then, *dominance* for query *q* is:

19 $$\begin{aligned} A_q(D_1, D_2)&= \frac{\left( \frac{ ranksum }{|\delta _q^1(D_1, D_2)|} - \left( |\delta _q^1(D_1, D_2) + 1\right) /2\right) }{|\delta _q^2(D_1, D_2)|} \end{aligned}$$

The results for dominance are shown in Table 5 for the top $N=10$ retrievals, respectively. The top-most subtable shows the average dominance (on average how often does one descriptor perform better than another), and the middle subtable shows the percentage of queries where one descriptor performs better than another. The bottom-most subtable shows how often one descriptor has a “big win” over another descriptor, by which we mean the percentage of queries for which a descriptor has a dominance ${\ge }{0.8}$ over the descriptor to which it is being compared.

From Table 5 we can see that when features in the SO group are compared to each other, the dominance scores are are all approximately 50%. For these descriptors, if you choose two at random you have about an equal chance of getting the most accurate return set for a given query image.

For the IO group there is a gradual progression of L–A–S dominating the other three, with the smallest difference being between L–A–B and L–A–S. When we compare descriptors in the IO group with those in the SO group, we find that those in the SO group dominate approximately 60% of the time. However, considering “big wins” (Table 5b), we find that the SO group query results rarely dominate the IO group query results with a dominance of more than 0.8. That is, while the SO group on average produces results with more metadata matches than the IO group, it is rare that the SO consistently produces many more metadata matches. This tends to indicate that the IO descriptors are returning a comparable set of results to the SO group results but with a different set of images that are less focused on matching the metadata precisely.

**Table 5:** Average dominance, frequency of dominance, and frequency of “big wins” for top 10 retrievals

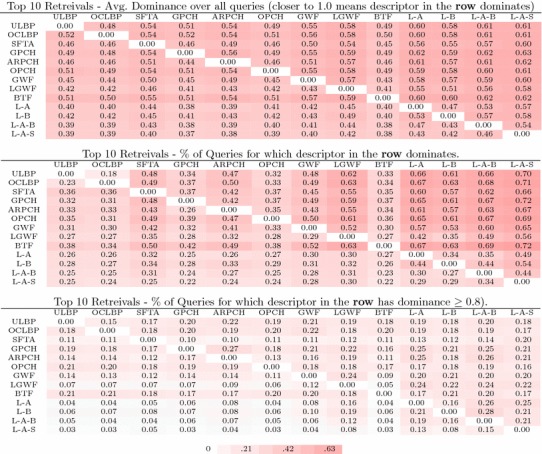	

### Discussion and conclusions

Our results have built a robust picture of the strengths and weaknesses of the different descriptors. Considering overall accuracy, it does appear that the descriptors perform largely the same in the way that they match images, with less than a percentage point of accuracy between the top 5 methods. This means that if we were worried strictly about finding as close to perfect matches as we can, then any of these descriptors would likely be adequate. This is particularly true when there are small numbers of images in the sets that have exact matches against the ground truth metadata. For example, if there are only 7 objects that perfectly match in the ground truth to the query image, we can be relatively certain each of the algorithms will return those 7 reliably.

However, given that we are looking to support online search, we are interested in more than just exact matches. Researchers working with image archives rely on comparing and contrasting different, yet related, objects together while making their decisions. As a result, it was important to understand whether each descriptor was just returning the same sets as every other descriptor or whether there were distinct differences in their performance. There was distinct difference between the best and worst overall performing descriptors (the SO group and the IO group). First, it is interesting that in the SO group there is almost no variation in the sets of images they retrieve. Even at their most varied, we are only seeing about 13% variation in the images that are returned from the standard descriptors, with most hovering around only 8% difference. This tends to imply that if you choose any one of these algorithms, you are going to get largely the same set of images being returned.

In comparison, the IO group of descriptors retrieves image sets that are 20–30% different from the SO group. It appears that the IO descriptors are better at detecting differences in material while trading off shape matching accuracy, whereas the SO descriptors are doing the opposite. If we are working with a single collection of flint, where most pieces come from the same source stone, then the SO descriptors are likely to perform better. However, for a more varied collection of bifaces, comprised of flint, glass, etc., we would expect the IO descriptors to be superior.

Considering the dominance between the different descriptors, it appears that one could choose one of the SO descriptors at random and produce largely the same results over a series of query images. However, choosing one of the IO descriptors would result in a system that returns slightly more varied results. This may be important if search system designers want to encourage serendipity in their search, something which is often valued by researchers.

In future work, we will pursue the evaluation of these different descriptors with users in the archaeological archives. It would be particularly interesting to know which, if any, descriptor is preferred by users in supporting their search.

Overall, the new fused descriptors proposed in this paper have a set of advantages and disadvantages over the descriptors in the SO group. In general, the new descriptors appear to work well in finding relevant and related images in an image search. However, they have the advantage of producing more varied sets of results which may add value to search systems, in particular in heterogeneous image archives where there will be large variation in material qualities of artefacts.
